# Thirty years of cutaneous leishmaniasis in Tadla-Azilal focus, Morocco

**DOI:** 10.1016/j.parepi.2019.e00091

**Published:** 2019-01-31

**Authors:** Fatima-zahra Abou-Elaaz, Aboubakre Outourakht, Souad Bouhout, Oumnia Himmi, Souad Guernaoui

**Affiliations:** a Laboratory of Biotechnology and Valorisation of Plant Genetic Resources, Faculty of Sciences and Techniques, University of Sultane Moulay Slimane, Beni Mellal, Morocco; b Geophysics, Natural Patrimony and Green Chemistry Research Center (GEOPAC), Geo-Biodiversity and Natural Patrimony Laboratory (GEOBIOL), Scientific Institute, Mohammed V University, Rabat, Morocco; c Directorate of Epidemiology and Disease Control, Ministry of Health, Rabat, Morocco

**Keywords:** Cutaneous leishmaniasis, Age structure, Space-time distribution, Altitude, Tadla-Azilal, Morocco

## Abstract

Cutaneous leishmaniasis (CL) due to *Leishmania tropica* is a major health problem in Tadla-Azilal focus, Morocco, where the first case was registered, here, in one locality named Tanant in 1987. So far, CL remains endemic and largely widespread. The objective of this study was to analyze the current eco-epidemiological situation after thirty years of active transmission. Data used are the official ones, obtained from the Moroccan Ministry of Health.

Between 1998 and 2015, 5518 CL cases were registered in three provinces; Azilal, Béni Mellal and Fquih Ben Salah. CL has spread, from the historical focus in Azilal along two axes; one to the Northeastern Mountains and the other to the Northwestern plains.

CL infected both genders and all ages, with large number of women (53%) and children (75% had <9 years old). More interestingly, age range at risk was larger within females than males, and this difference was more pronounced in Fquih Ben Salah where the disease had newly emerged. Similarly, age ranges at risk were larger and fluctuated significantly each year particularly in new emerging areas in Béni Mellal and Fquih Ben Salah. All these variations may testify, at least in part, to the process of acquiring immunity.

Altitude structured CL spatiotemporal distribution. CL was more prevalent in two altitudes ranges; 400–500 m and 800–900 m. The situation and duration of period of diagnostic of CL varied largely according to the altitude. These different scenarios could be related to the seasonal dynamics of vector populations.

## 1 Introduction

Leishmaniasis is a parasitic vector-borne disease, caused by the flagellate protozoa of the genus *Leishmania* (Trypanosomatida, Trypanosomatidae) (Reithinger et al., 2007), and transmitted by the bite of an infected female of sandflies (Diptera, Psychodidae) (Khezzani and Bouchemal, 2016).

In Morocco, cutaneous and visceral leishmaniasis are endemic and are classified in the list of compulsory-declared diseases (ministerial order n ° 683–95 from March the 3th, 1995). Cutaneous leishmaniasis (CL) is a major public health problem, where 2813 cases were recorded in 2015 (Moroccan Ministry of Health, 2016; Kahime et al., 2016). It's due particularly to *Leishmania tropica* and *L*. *major* (Moroccan Ministry of Health, 2016). Infections due to a dermotropic variant of *L*. *infantum* also have been found (Rioux et al., 1996; Lemrani et al., 1999). CL due to *L*. *tropica* is transmitted by *Phlebotomus sergenti* (Guilvard et al., 1991; Pratlong et al., 1991). The cycle is considered to be anthroponotic despite the parasite being isolated from dogs (Dereure et al., 1991). In 1989, the first hypo-endemic rural focus of CL due to *L*. *tropica* was diagnosed in Central Morocco (Marty et al., 1989), where the first case was notified in one locality named Tanant (Azilal province). Other comparable foci were identified in semi-arid areas in Smimou (Pratlong et al., 1991), Taza (Guessous-Idrissil et al., 1997) and Zouagha Moulay Yaacoub (Rhajaoui et al., 2004). Since then, CL was epidemic in Chichaoua (Guernaoui et al., 2005), and recently in Sidi Kacem (El Miri et al., 2016).

After the first case diagnostic in Tadla-Azilal region, CL still, after 30 years, considered as a major health problem. 3916 CL cases registered between 2009 and 2015 according to the data base of the Direction of Epidemiology and the Disease Control, Rabat, Morocco.

The objective of this paper is to analyze the current epidemiological situation after thirty years of active transmission, to extract the main factors that manage the spatio-temporal distribution of the disease and also to interpret the CL spread in this large focus and in neighboring regions.

## 2 Materials and methods

### 2.1 Study area

Tadla-Azilal is situated in Central Morocco, between 32.0043 N and 6.5783 W, over an area of 17,125 km^2^ (Fig. 2). Administratively, this region is divided into three provinces; Azilal, Béni Mellal and Fquih Ben Salah and 16 municipalities; 9 from urban and 7 from rural areas (Fig. 2). The total population from this region was about 1,516,200 people in 2012 representing 4.65% of total population in Morocco (High Commission for Planning Morocco, 2013). Agriculture is the most important activity, followed by tourism and trade.

Tadla-Azilal region has a high ecological and climatic diversity due to its location between the two important Moroccan mountains ranges: the High and the Middle Atlas. The climate varied from semi-arid to humid. The averages annual temperature show significant differences; they vary between a maximum of 40 °C in Béni Mellal province and a minimum of 2 °C in Azilal province. The average rainfall is 100 mm in arid zones and 600 mm in wetlands. The vegetation is principally forester, occupies a 396,743 ha area. The pins, juniper, holm oak and thuya are the main natural forest species (Taïbia et al., 2015).

### 2.2 Data collection

Data presented here are the official ones, obtained from the Moroccan Ministry of Health (MMH), Direction of Epidemiology and the Disease Control, Rabat, Morocco. CL patients were diagnosed clinically, and the diagnostic was confirmed by officials in hospitals and health services in Tadla-Azilal region.

As the obligation to declare the disease was effective in Morocco from 1998, the information available, from 1998 to 2008, included only the number of CL cases in each region. From 2009, the patient file was more detailed; and contained information about sex, age, time of diagnostic (year and month) and geographical location (province, sanitary sector and locality).

### 2.3 Data analysis and mapping

Data were entered in a Microsoft Excel 2010 file. The χ^2^ test was used for comparison of categorical variables. For all test, the significance level was 0.05. The Student *t*-test used to compare between the mean numbers of CL cases in different altitude ranges. Box plots were prepared and the correlation report was calculated for comparing age structure in different provinces for both sexes and also for each year.

We studied the spatio-temporal dynamics of CL (dissemination of *Leishmania* parasite) by mapping the distribution of cases, each year, in the different provinces, using ArcGIS software (ArcGIS 10.2.2).

## 3 Results and discussions

In this large focus, The ITS1 PCR-RFLP was used and identified *L*. *tropica*: from patients in Foum Jamâa, and from *P*. *sergenti* in Azilal (Arroub et al., 2013; Ajaoud et al., 2015).

**Fig. 1:**
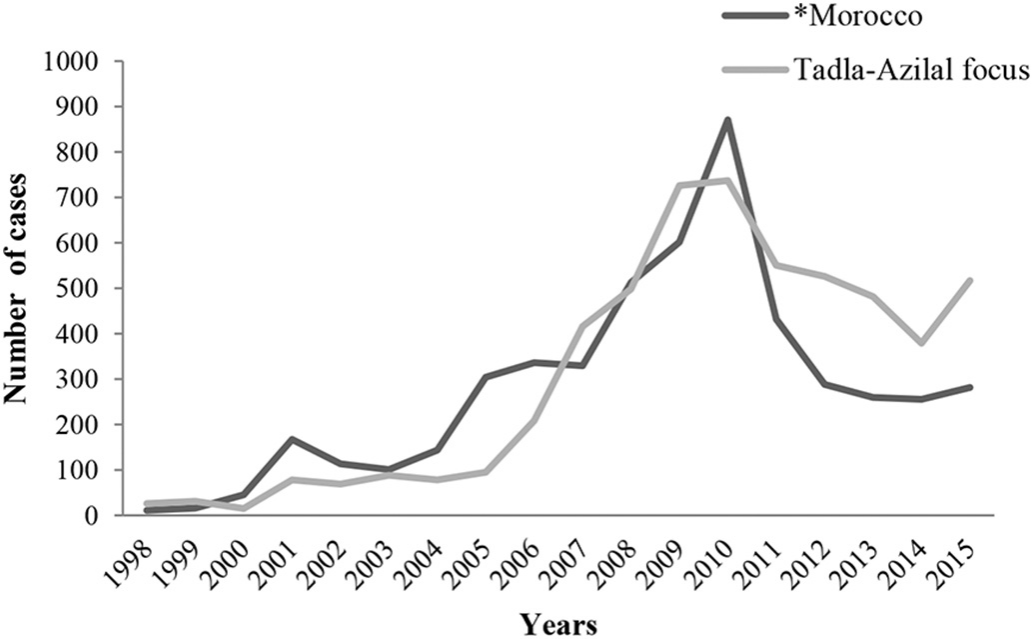
Cutaneous leishmaniasis in Morocco and in Tadla-Azilal focus between 1998 and 2015. (Data source: Moroccan Ministry of Heath, 2016). ^⁎^Number of cases multiplied by 10^−1^.

According to data registered by the Direction of Epidemiology and the Disease Control, Rabat, Morocco, between 1998 and 2015, 5518 cases of CL were registered in Tadla-Azilal focus, corresponding to 10.89% of total cases in Morocco (Fig. 1). In general, for this same period, the annual evolution of number of CL cases in Tadla-Azilal focus, is proportionally correlated with than in Morocco (Fig. 1). During the first 12 years (1998, 2010), the number of CL cases increased significantly, and passed from 26 in 1998 to 737 in 2010. A significant decrease was registered between 2011 and 2014. The CL number passed from 550 to 379, and in 2015, we observed that CL progresses again. The difference being statistically significant (χ2 = 856.82).

### 3.1 Evolution of spatio-temporal distribution of CL

Table 1 shows the most important sanitary sectors with CL from 2009 to 2015 in Tadla-Azilal focus. Results demonstrated the large spatial distribution of the disease. In this region, according to administrative subdivision, CL is registered in three provinces; Azilal, Béni Mellal and Fquih Ben Salah. The latest situation registered in 2015 declared the presence of CL in 25 sectors and 210 localities.

**Table 1:** Number of CL cases in the three provinces of Azilal, Béni Mellal and Fquih Ben Salah between 2009 and 2015.

Province	Sanitary sectors	Years							Total
		2009	2010	2011	2012	2013	2014	2015	
Azilal	Afourer	145	61	44	42	51	51	39	433
	Ait Attab	5	16	11	5	10	32	13	92
	Ait Majden	8	16	1	5	3	0	9	42
	Azilal	5	9	12	3	0	0	0	29
	Bzou	85	90	45	67	90	62	50	489
	Bni Hassane	53	62	26	23	11	0	19	194
	Bin Elouidan	11	8	8	8	2	0	2	39
	Beni Ayyat	35	2	0	0	0	0	0	37
	Demnate	2	8	4	6	1	0	4	25
	Foum jamaa	41	51	33	40	58	69	94	386
	Timoulilte	28	21	19	14	1	0	7	90
	Tabia	10	29	17	25	4	0	24	109
	Tiski	20	50	40	21	12	0	35	178
	Taounza	4	7	17	18	9	0	9	64
	Tilouguite	2	0	13	4	4	0	1	24
	Tibihite	3	0	0	10	0	0	0	13
	Ouaouizrt	87	23	19	19	26	28	4	206
	Tanant	33	38	35	31	23	21	26	207
	My Aissa Ben Driss	5	0	1	0	0	0	0	6
	Sidi Ali Ben Brahim	0	4	1	4	4	0	18	31
	Tanfarda	0	12	24	6	1	0	3	46
	Tidli	0	28	6	2	6	6	0	48
	Tislit	0	11	0	5	0	0	0	16
	Bouchiba	0	0	10	2	5	0	4	21
Béni Mellal	Foum oudi	0	0	14	11	19	0	0	44
	Foum Al-Anser	7	16	9	6	7	1	5	51
	Béni Mellal	13	17	22	9	13	6	10	90
	Fariyata	1	3	1	8	1	3	4	21
	Tagzirt	2	7	3	7	4	8	2	33
	Tanougha	7	11	10	12	13	8	12	73
	Tadla	1	0	0	1	0	0	0	2
	Zaouiat cheikh	47	19	17	26	43	19	31	202
Fquih Ben Salah	Ouled Ayyad	16	5	14	29	40	32	33	169
	Had Boumoussa	0	1	1	0	0	6	4	12
Total		676	625	477	469	461	352	462	3522

Diversity of epidemiological profiles is shown in Fig. 3. Furthermore, in 2009 (Fig. 3A), CL was registered in Azilal province. Here, the disease was largely widespread, concerned 32 sanitary sectors and 195 localities (703 cases). Epidemiological situation was stable. Moreover, the disease is well installed in this area where the transmission is assured for 30 years. Moreover, the first case of CL due to *L*. *tropica* recorded was registered here, precisely in Tanant in 1987 (Marty et al., 1989).

In 2009 (Fig. 3A), CL emerged for the first time in Béni Mellal province. Here, the disease is specifically registered in mountainous areas from Béni Mellal city to Zaouiat Cheikh (716 m a.s.l.).

The propagation still continuous and in 2010 (Fig. 3B), from Azilal region to Northwestern localities in Fquih Ben Salah province (Had Boumoussa, Souk Sebt, Oulad Ganou and Oulad Illou) and to Southeastern mountainous localities (Tidili, Anzou, Ait Mazigh, Ait Tanlil and Ait Bougumez).

The situation stills stable in 2011 and in 2012, in three provinces of the region (Fig. 3B). CL propagation remained continuous in the two precedent axes, with the emergence of the disease in the urban area of Fquih Ben Salah city, and in some High-mountains region at Zaouiat Ahansal (1629 m), Aghbala (1715 m) and Agouti (1810 m) (Fig. 3C, D).

The period between 2013 and 2014 marked a phase of regression with a net and significant decrease (χ^2^ = 10.08) in the number of CL cases. The presence of the disease in endemic areas was remarkable in 36 sectors in 2013 and 20 sectors in 2014; notably in Bzou, Tanant, Afourer, Foum Jamaa and Ait Attab (Fig. 3E, F).

In 2015 (Fig. 3G), CL started again and recolonized the former endemic areas with a clear increase in the number of cases (512 cases).

Considering the space-time dynamic of CL in this large focus, our data are analyzed comparing the situation in Azilal province, where the disease is present since 1987, in Béni Mellal and in Fquih Ben Salah provinces, where CL emerged recently, in 2009 and 2011, respectively (Fig. 3, Table 1).

### 3.2 Evolution of age structure of both genders according the time and space

In general, in Tadla-Azilal focus, both genders are concerned by CL; females were more affected (53% of total cases). The overall male: female ratio was 0.9, and each year, this difference was statistically significant (χ^2^ = 7.58, p-value < 0.05).

This result is in agreement with those reported in others Moroccan foci, notably in Taza (Guessous-Idrissil et al., 1997), Taounat (Chiheb et al., 1999), Chichaoua (Guernaoui, 2006), Al Haouz (Ramaoui et al., 2008), Sidi Kacem (El Miri et al., 2016), and other countries as in Southern Iran (Khosravani et al., 2016). In contrary, in other studies, males have a higher probability to be affected by CL in general than females like in the Islamic Republic of Iran and in Algeria (Shirzadi et al., 2015; Khezzani and Bouchemal, 2016).

The number of cases by age group is shown in Fig. 4. Patient's age ranged from 1 to 90 years old. Mean ± Standard deviation and median ages were 51.58 ± 23.71 and 4 years, respectively. 75% of CL cases have <9 years old, demonstrating that children are the most important population at risk in this focus. The same result was registered in Chichaoua and in Al Haouz Moroccan foci (Guernaoui, 2006; Ramaoui et al., 2008) and was explained by the peri-domiciliar ability of *P. sergenti* to transmit *Leishmania*. In Spain, 56% and 30% of cases were children <5 years or <14 years respectively in Granada and in Toledo (Alcalde et al., 1989; Urrutia et al., 2000). The high percentage of children could be explained by immune system immaturity and there sensitivity to infection (Özkeklikçi et al., 2016; Shirzadi et al., 2015).

More interestingly, CL infected both genders and all age groups, with large number of women and children. The age structure was different according to the gender (Fig. 5). Age range at risk was larger within females than males, and this difference was more pronounced in Fquih Ben Salah province where the disease had newly emerged. Indeed, the correlation report between gender and age was 0.15, 0.17, and 0.23 in Azilal, Béni Mellal and Fquih Ben Salah provinces, respectively.

Similarly, data analysis (Fig. 6) showed that the age range at risk varied significantly each year. In Azilal, the range of ages affected was relatively the same. In Béni Mellal and Fquih Ben Salah where CL had newly emerged, age ranges were larger and fluctuated significantly. The correlation report between age and year was 0.28 in Azilal, 0.41 in Béni Mellal and 0.39 in Fquih Ben Salah.

The variation of age range decreased after each year in Azilal province, and it could be due to acquisition of the system immune since the disease is old in this province. Furthermore, in Béni Mellal and Fquih Ben Salah provinces, the large variation of age range could be due to the recent installation of CL in these areas.

### 3.3 Altitude and spatio-temporal distribution of CL in Tadla-Azilal focus

**Fig. 2:**
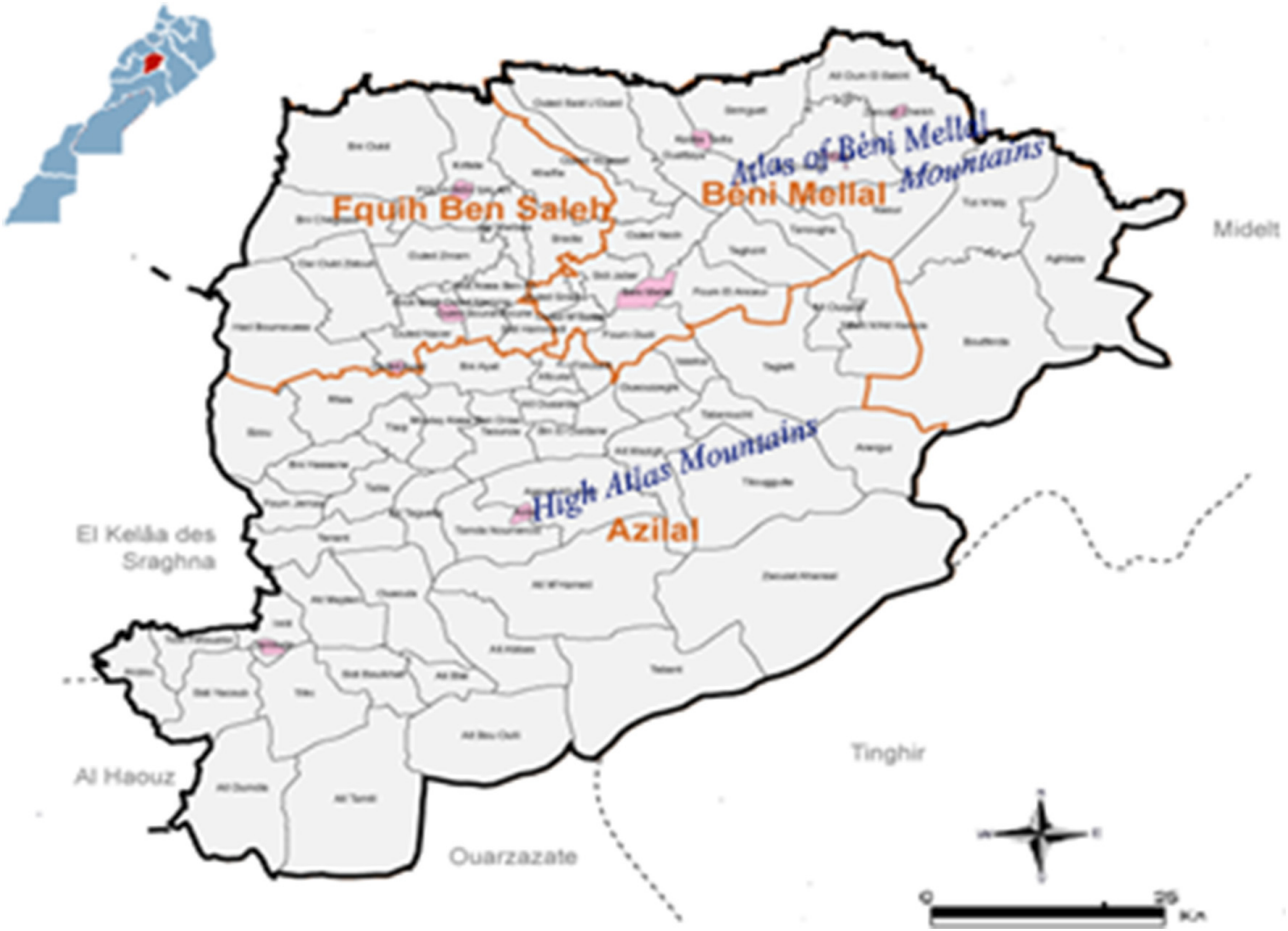
Map of Tadla-Azilal region showing the different provinces with CL cases.

In this large focus, the relief showed being an important ecological factor. Furthermore, Azilal province, the historical focus of *L*. *tropica* in Morocco, is situated in High-Atlas Mountains. While in Béni Mellal (507 m) - Zaouiat Cheikh (716 m) placed in Atlas of Béni Mellal, this latter marked the transition between the High-Atlas and Middle-Atlas Mountains in Morocco (Fig. 2).

**Fig. 3:**
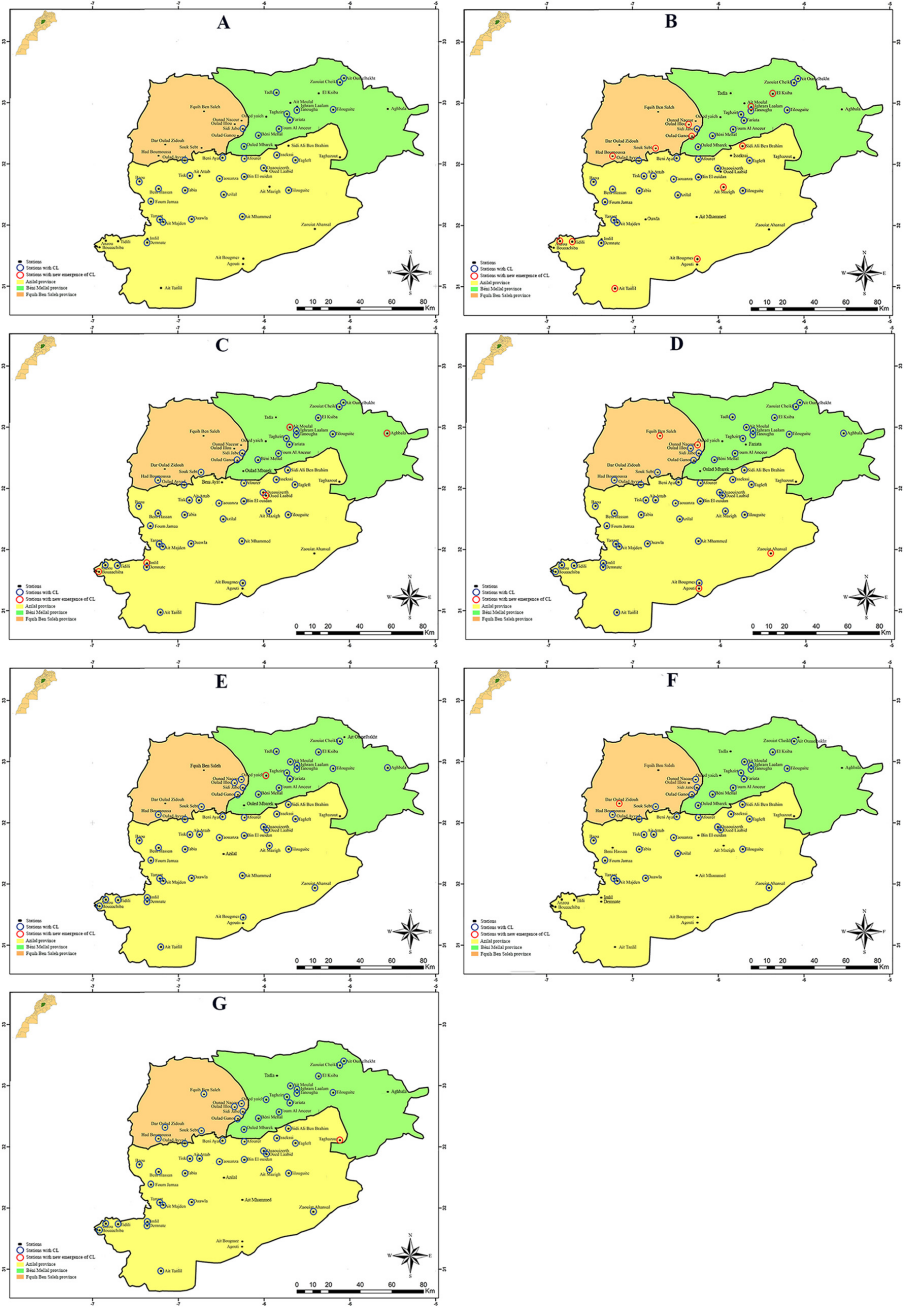
Spatio-temporal dynamics of CL cases in Tadla-Azilal from 2009 to 2015.

**Fig. 4:**
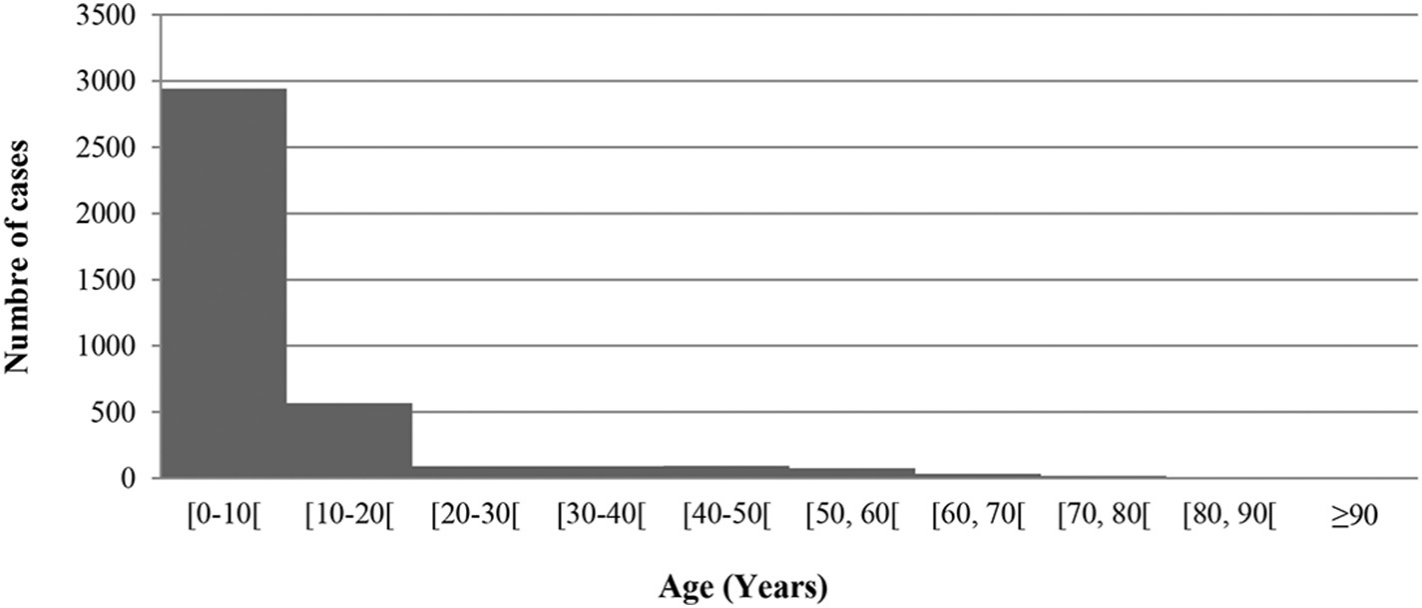
CL patient's ages in Tadla-Azilal focus from 2009 to 2015.

**Fig. 5:**
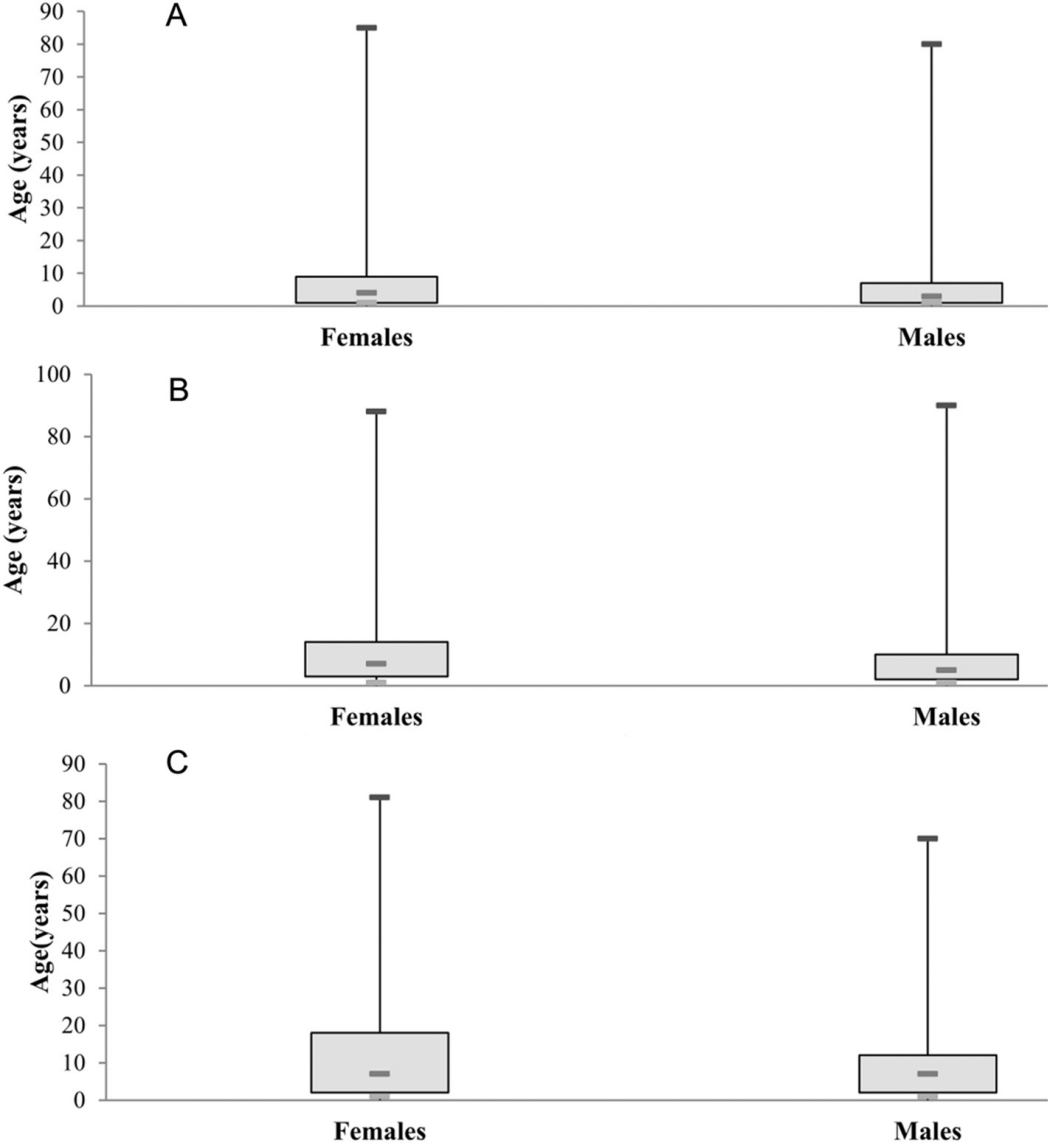
Box plots of age within males and females in Azilal (A), Béni Mellal (B), and Fquih Ben Salah (C) provinces from 2009 to 2015.

**Fig. 6:**
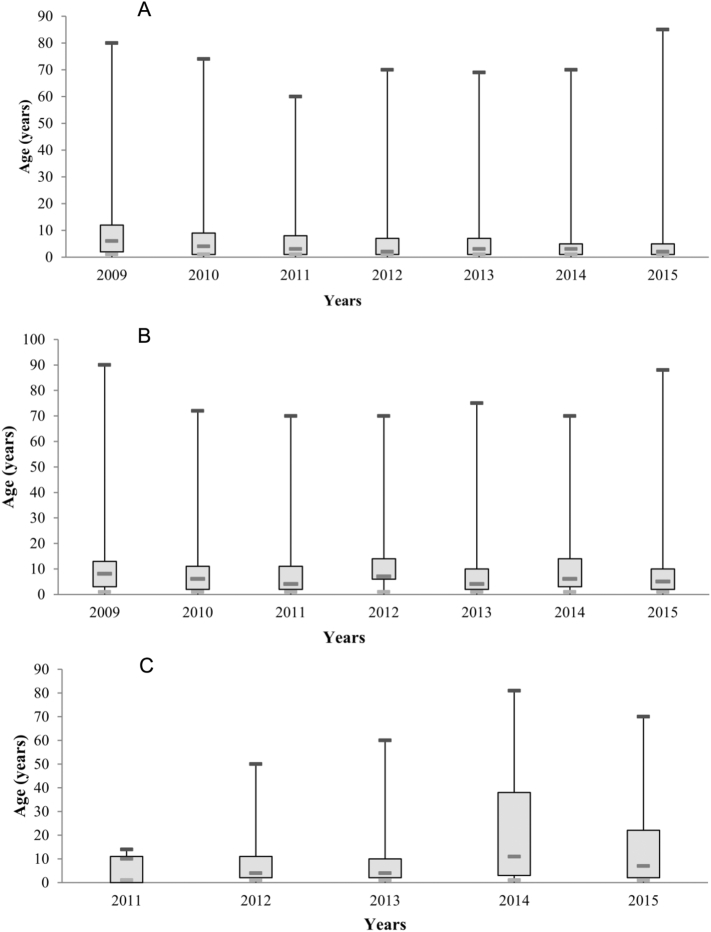
Box plots of age according to the year in Azilal (A), Béni Mellal (B), and Fquih Ben Salah (C) provinces from 2009 to 2015.

**Fig. 7:**
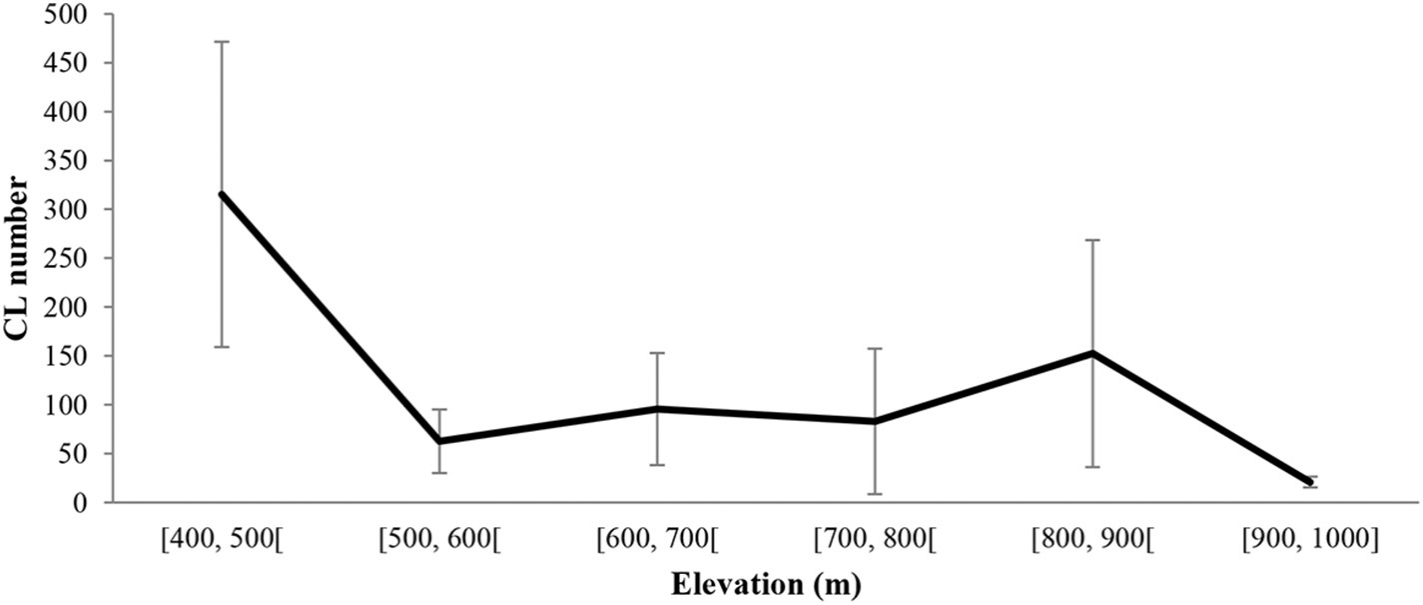
Mean annual number of CL cases (±SD) in different altitude ranges in Tadla-Azilal focus from 2009 to 2015.

**Fig. 8:**
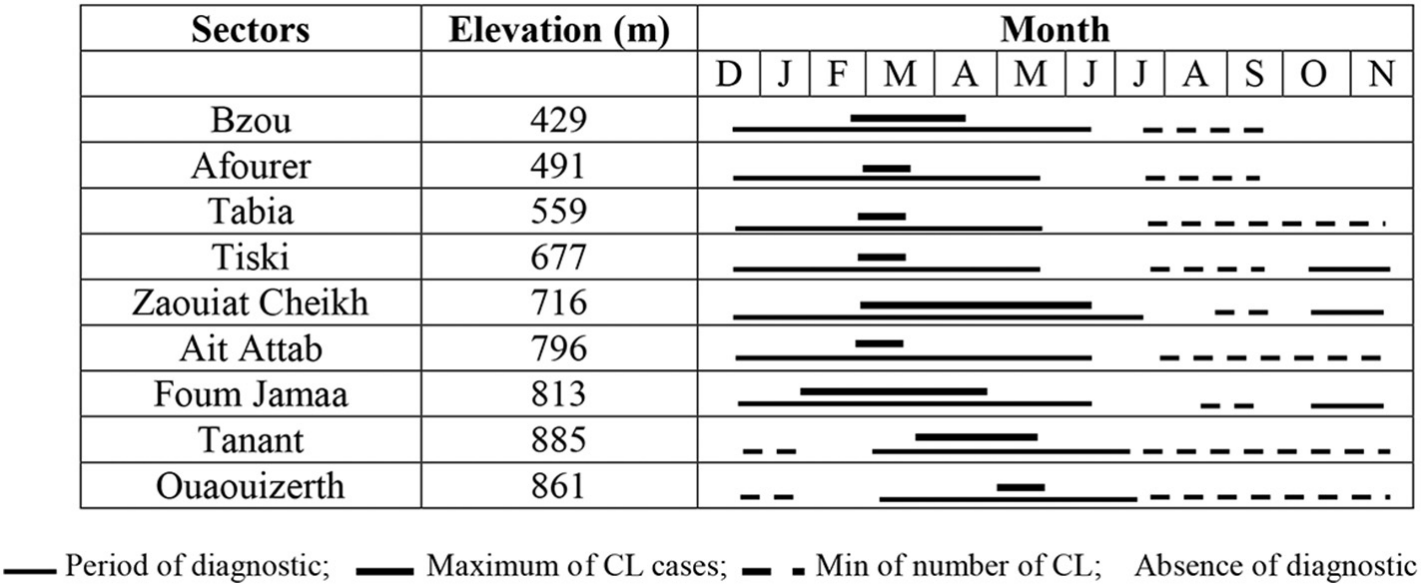
Months of diagnostic of CL patients in different altitudes in Tadla-Azilal focus from 2009 to 2015.

Two zones were highly prevalent for CL (Fig. 7). The first one, situated between 400 and 500 m a.s.l., and where the maximum mean number of CL was reported (315.33 ± 156.07). The second one, with altitudes varying from 800 to 900 m a.s.l. Here, an average of 152.40 ± 116.11 was registered. CL was also recorded from stations between 500 and 800 m a.s.l., with CL mean number varying from 62.80 to 95.75 cases (Fig. 7). The difference was statistically significant between altitude from 400 to 500 m a.s.l. and from 800 to 900 m a.s.l. (*t* = 0.036).

In the same manner, altitude seemed influencing the period of diagnostic of CL in this focus (Fig. 8). This period was long, 9 to 10 months, in localities with altitudes ranging from 500 to 800 m a.s.l., notably in Tiski, Zaouiat Cheikh, and Foum Jamaa. Here, the cases started to appear in October and this continued until June–July (Fig. 8).

On the other hand, this period was relatively short, earlier from December to Mai-June (6 month) as in Bzou (429 m) and Afourer (491 m), and later, from February to June each year (5 month), as in Tanant (885 m) and Ouaouizerth (861 m) (Fig. 8).

These different scenarios must, at least in part, be related to the seasonal dynamics of vector populations in these regions. Moreover, in the High-Atlas Mountains, Morocco, the spatial distribution and seasonal activity of vectors are structured according to altitude (Guernaoui et al., 2006). The question of the existence of other vectors than *P. sergenti* must also be tested.

## 4 Conclusion

Tadla-Azilal region, after 30 years of CL active transmission, still a divers and complex focus. The current situation shows that the problem persists and increases. The disease spreads in space along two axes; one to the Northeastern Mountains and the other to the Northwestern plains areas. Various CL distribution models, in time and in space, are structured by the altitudinal zones. And this makes the control very difficult in practice. Serious studies of vector populations should be undertaken for better programming CL control in this focus and for limiting its spread in neighboring areas.
